# Characterization and Toxicity of Hypoxoside Capped Silver Nanoparticles

**DOI:** 10.3390/plants11081037

**Published:** 2022-04-11

**Authors:** Umar M. Badeggi, Sylvester I. Omoruyi, Enas Ismail, Charlene Africa, Subelia Botha, Ahmed A. Hussein

**Affiliations:** 1 Chemistry Department, Cape Peninsula University of Technology, Symphony Rd., Bellville 7535, South Africa; umbadeggi@ibbu.edu.ng (U.M.B.); omoruyis@cput.ac.za (S.I.O.); 2 Department of Restorative Dentistry, Faculty of Dentistry, University of the Western Cape, Cape Town 7505, South Africa; 3 Department of Medical Biosciences, University of the Western Cape, Robert Sobukwe Rd., Bellville 7535, South Africa; cafrica@uwc.ac.za; 4 Electron Microscope Unit, University of the Western Cape, Bellville 7535, South Africa; subotha@uwc.ac.za

**Keywords:** *Hypoxis hemerocallidea*, hypoxoside, antimicrobial, cytotoxicity, silver nanoparticles, characterization, glioblastoma cells

## Abstract

The reducing potential of plant extracts in the green synthesis of nanoparticles has been associated with their phytochemicals. Although pharmacologically inactive, a norlignan diglucoside “hypoxoside” (HP) occurs in large quantities in the extract of *Hypoxis hemerocallidea* (HE). In this work, HP was isolated from HE where both were used in the biosynthesis of the corresponding silver nanoparticles (HP-AgNPs and HE-AgNPs). The AgNPs were fully characterized using various physicochemical techniques and their antimicrobial and anticancer properties were evaluated. Transmission electron microscopy (TEM) revealed sizes of 24.3 ± 4 nm for the HE-AgNPs and 3.9 ± 1.6 nm for the HP-AgNPs. The HE-AgNPs demonstrated enhanced anti-bactericidal effects on *Escherichia coli* and *Salmonella enterica* with a minimum inhibitory concentration (MIC) value of 1.95 µg/mL, competing well with the standard drug. The cytotoxic activity showed that the HE-AgNPs reduced cell viability with an IC50 of 0.81 and 4.0 µg/mL, respectively, for the U87 and U251 cells, while the HP-AgNPs displayed 0.20 and 0.55 µg/mL for both cell lines, respectively. Furthermore, while the HE-AgNPs were selective to U87 alone, the HP-AgNPs were selective to both glioblastoma cells tested. The study demonstrated the ability of a single phytoconstituent (hypoxoside), not only as the chief bioreductant in the extract, but also as a standalone reducing and capping agent, producing ultra-small, spherical, and monodispersed AgNPs with enhanced biological properties.

## 1. Introduction

The physical and chemical-based synthesis of metallic nanoparticles has limited benefits in biomedical applications due to several disadvantages [1]. Major among them is the use of toxic chemicals, which not only limit the applicability of the nano products but also have a negative impact on the ecosystem [2]. Physical protocols are also faced with demerits such as the need for expensive and sophisticated equipment that is unavailable in most laboratories [3]. Biological or green synthesis procedures present a better alternative as the nanomaterials are biocompatible and can be applied in various fields, including in water purification and biomedical fields [4]. Interestingly, silver nanoparticles are arguably the most researched metallic nanoparticles. Recently, pure natural compounds have also been used in the green synthesis of nanoparticles [5] using tannic acid [6], quercetin, and gallic acid [7]. Silver nanoparticles have been reported to possess anticancer [8], antioxidant [9], and antimicrobial [10] properties.

The characterization of nanoparticles is of the utmost importance. The characteristic features of the hybrid particles, such as the size, shape, and crystallinity, need to be ascertained before onward applications. This is because certain applications are size- or shape-dependent. Thus, these have been determined through techniques such as ultra-violet visible spectroscopy, electron microscopy, dynamic light scattering, selected area electron diffraction, and X-ray diffraction [10,11].

Renewed attention has been given to silver nanoparticles for biological applications because of the ease of their surface chemistry and biocompatibility. For instance, antibiotic resistance has been a global challenge for a long time, affecting both the health and economy of the world [11]. Therefore, in search of alternatives, green nanotechnology has drawn attention as metallic nanoparticles (MNPs) have displayed inhibitory activities against many pathogenic bacterial species [12]. Silver nanoparticles have been used as a top antibacterial agent against various bacterial species because of their unique characteristics. Additionally, AgNPs have shown anticancer activity when synthesized using plant materials [9]. Several researchers have reported AgNPs with both antimicrobial and cytotoxic properties when naringenin, hesperidin, diosmin, gallic acid, and quercetin were used as reducing agents [13,14].

*Hypoxis hemerocallidea* (HE) belongs to *Hypoxidaceae*, with a genus of about 90 species, of which 29 are native to South Africa (SA). The plant is widespread in many areas of Southern Africa. It stretches into Zimbabwe, Mozambique, and parts of East Africa [15]. It can be identified by features such as the unique “star-like” yellow flowers. In SA, the plant is locally known as “sterblom” by the Afrikaans community, while the isiZulus call it “inkomfe” and the Sesothos call it “lotsane”. The English name, which is the most popular, is African potato [15]. Extracts of HE have been traditionally employed in the management of diseases such as cancer, HIV/AIDS, urinary infections [16], acne, dysentery, dermatitis, wounds, dizziness, testicular tumours, bladder disorders, and high blood pressure [17,18]. The extracts of HE were found to possess anti-inflammatory, antibacterial, antioxidant, and antifungal activities [17,19]. To date, only a few compounds have been isolated from this plant. They include sterols and a major constituent, hypoxoside [20,21], believed to be responsible for the medicinal properties of the plant.

Despite the huge medicinal value of this plant, little is known about its ability in modern-day green nanotechnology research. Previously, our group reported the use of hypoxoside in gold nanoparticles synthesis and evaluated their immunomodulatory effects in macrophages and natural killer cells as well as their wound healing potential [22,23]. The ability to synthesize other important nanoparticles such as platinum and silver has not yet been studied. Moreover, silver nanoparticles possess superior activities in applications such as antimicrobials compared to gold and other metals [24]. Therefore, in this study, the biofabrication of AgNPs using HH extract and hypoxoside was undertaken. A detailed explanation of the involvement of hypoxoside in the green synthesis of the AgNPs was provided for the first time. The variation in the biological activities between the extract- and hypoxoside-mediated AgNPs was also examined.

## 2. Results and Discussion

### 2.1. Isolation, Characterization, and Properties of Hypoxoside

**Figure 1:**
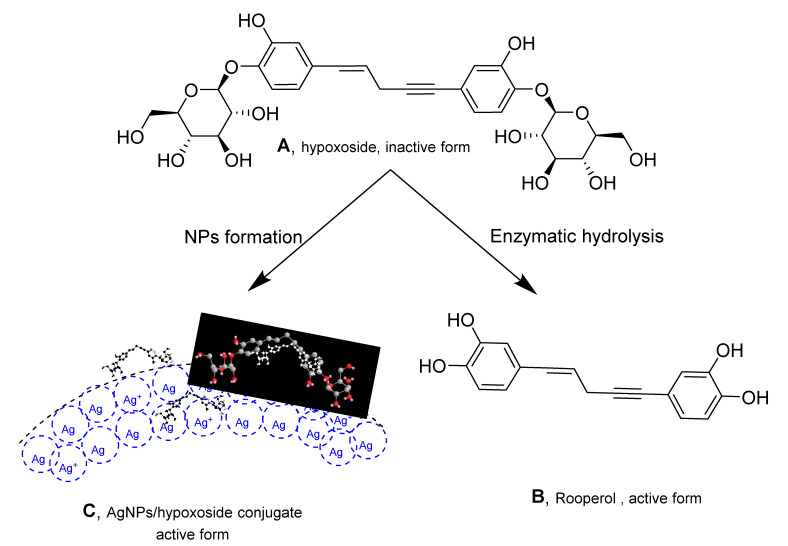
Structure of hypoxoside (**A**) and rooperol (**B**) upon conversion using β-glucosidase enzyme and hypoxoside/AgNPs conjugate (**C**).

Hypoxoside, a glycosylated norlignan that is the major compound in the corn of *Hypoxis hemerocallidea*, is named (*E*)-1,5-bis(4′-β-d-glucopyranosyloxy-3′-hydroxyphenyl)-pent-4-en-1-yne [25]. Its isolation from HE extract has previously been reported [26] and the obtained spectra correlate with those of the published literature. Hypoxoside has a low toxicity with an uncommon aglycon structure. It is composed of two glucose units at the edges of the two benzene rings in the pentenyne skeleton (Figure 1A) [15]. Various reports on the compound associated its presence with many traditional uses of the plant [27]. However, hypoxoside was found to be pharmacologically inactive on its own but is usually converted to its aglycon, rooperol, through hydrolysis of the former by the action of a β-glucosidase enzyme in the human gut (Figure 1) [28]. Rooperol, on the contrary, has fascinating biological activities such as anti-inflammatory, anticancer [29] antibacterial, immunomodulatory, antioxidant, antitumor, and anti-convulsant activities [30]. Therefore, there is a need to find other ways by which hypoxoside can be more valuable, especially looking at its quantity from HH extract. One study has utilized hypoxoside in the synthesis of gold nanoparticles [22], but more can be achieved. Hence, this study aimed to use the hypoxoside in the green synthesis of AgNPs and to fully characterize it for the first time.

### 2.2. Biofabrication of HE-AgNPs and HP-AgNPs

**Figure 2:**
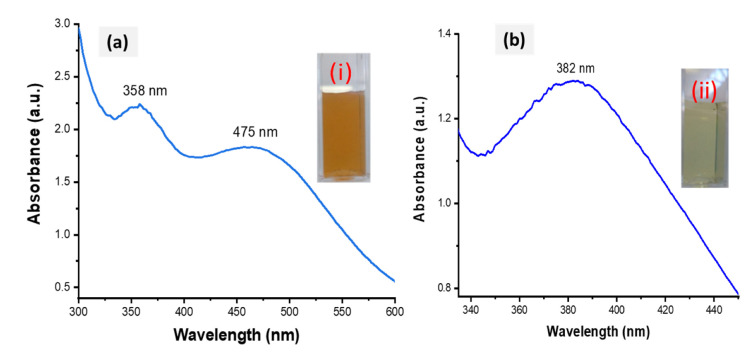
UV-Vis spectra of HE-AgNPs (**a**) and HP-AgNPs (**b**). The insets (i) and (ii) represent the colour of the HE-AgNPs and HP-AgNPs, respectively.

The success of the biofabrication of HE-AgNPs and HP-AgNPs was first observed upon a colour change to brown (Figure 2a) and pale yellow (Figure 2b), respectively. The total extract, HE, is composed of many biomolecules, each of which might have participated in the reduction of silver ions to form HE-AgNPs, while only one participated in HP, a solution of a single phytochemical (hypoxoside). The biofabricated HE-AgNPs showed absorbance at two regions (358 and 475 nm) (Figure 2a), whereas the HP-AgNPs displayed their maximum absorption at 382 nm (Figure 2b). The two SPR may be due to a mixture of shapes, while the HP-AgNPs, having an SPR of 382 nm, probably due to a single reducing agent, have a homogenous interaction mode with the Ag-salt [31,32].

The biofabricated HE-AgNPs showed absorbance at two regions (358 and 475 nm) (Figure 2a), whereas the HP-AgNPs displayed their maximum absorption at 382 nm (Figure 2b). The plasmonic resonance of AgNPs is sharp and more intense when compared to other metals such as gold because of the difference in the dielectric properties emanating as a result of the slight overlap between the surface plasmon resonance and the series of inter-band transitions in silver, which begins at 320 nm [33]. Similar plasmonic behaviour was reported by Sosa et al. [34], where two resonance bands between 320 and 500 nm were demonstrated. While they attributed the bands around 350 nm to spheres, Amendola and colleagues believed it to be due to the presence of cylindrical silver nanoparticles [33,34]. Nevertheless, the two SPR may be due to a mixture of shapes in the present study, as was later corroborated by the HRTEM image evaluation. Because of their symmetry, silver nanoparticles with spherical shapes possess only one plasmonic resonance [33,35]. This appeared to be the case with the HP-AgNPs having a surface plasmon resonance centred at about 382 nm. El-Naggar and colleagues reported AgNPs that absorb at about the same wavelength as our HP-AgNPs [36]. Also, using *Elaeagnus umbellate* extract, Ali and coworkers reported AgNPs with absorption at 398 nm [37]. Other researchers have reported silver nanoparticles whose plasmonic resonance falls between 300 and 800 nm [38,39]. The majority of the above authors also showed that the particles were mostly spherical. It is believed that mono- or multi-polar excitations may be dependent on certain features such as the nature of the material, the geometry, and the size of the nanoparticles being evaluated [34].

### 2.3. The Morphology and Size of HE-AgNPs and HP-AgNPs

**Figure 3:**
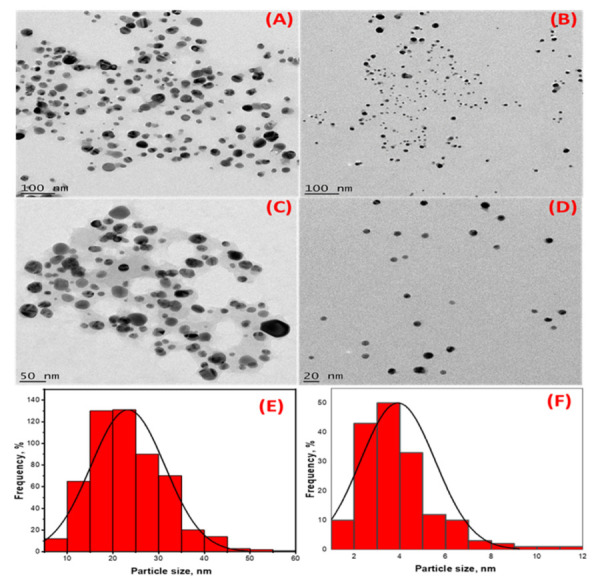
High-resolution transmission electron microscopy images showing the morphology of (**A**,**C**) HE-AgNPs and (**B**,**D**) HP-AgNPs. (**E**,**F**) are histograms displaying the particle size distribution of HE-AgNPs and HP-AgNPs, respectively.

In addition to the two SPR observed in the UV-Vis analysis, the TEM micrographs of the HE-AgNPs (Figure 3A,C) indicate a mixture of shapes, mainly spheres and a few cylindrical shapes. The nanoparticles also appear to aggregate. Previous studies [23] have reported various shapes when using an aqueous extract of HE in synthesizing gold nanoparticles. The presence of different phytoconstituents in the aqueous extract of HE might have contributed to the emergence of a mixture of shapes in the HE-AgNPs [40]. The HP-AgNPs were more monodispersed and mostly spherical (Figure 3B,D). This may be because of the purer nature of the reducing agent [41]. The mean particle sizes of 24.3 ± 4 nm and 3.9 ± 1.6 nm were obtained for the HE-AgNPs and HP-AgNPs (Figure 3E,F), respectively. This is in line with the particles present in the TEM micrograph. More aggregates were observed in the HE-AgNPs, thereby being responsible for the larger sizes. The UV-Vis analysis earlier suggested that the larger sizes might be due to mixture and aggregates. Although the spheres predominate (84.01%), there was a reasonable percentage (15%) of the cylindrical shapes. A small fraction of other shapes (cone) also made up about 0.93% (Table 1). The cylinders had a length of 2.26–20.58 nm, while the diameter ranged from 1.81 to 13.61 nm. The literature has demonstrated that nanoparticles of different sizes and shapes behave differently [42,43,44,45,46,47]. It is noteworthy that when single compounds/phytochemicals are employed in MNPs synthesis, small-sized and spherical-shaped NPs often result [41]. Hypoxoside therefore successfully mediated the green synthesis of quasi-spherically shaped silver nanoparticles with ultra-small sizes.

### 2.4. The Crystallinity of HE-AgNPs and HP-AgNPs

**Figure 4:**
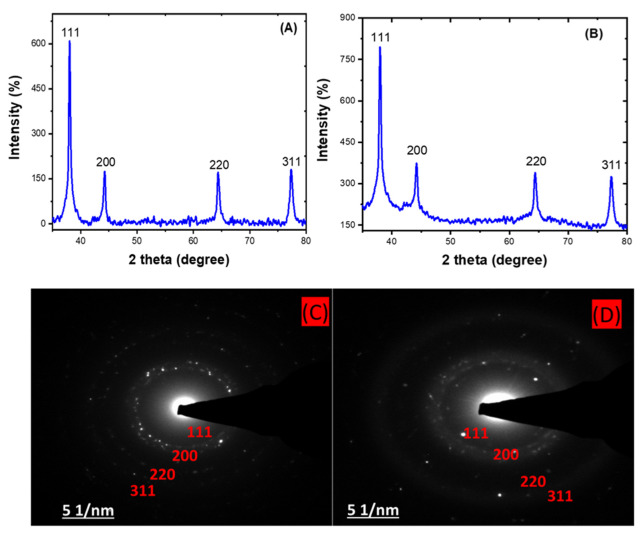
XRD patterns showing the diffraction peaks of HE-AgNPs (**A**) and HP-AgNPs (**B**). A selected area electron diffraction of HE-AgNPs (**C**) and HP-AgNPs (**D**) represented their crystallinity degree.

The X-ray diffraction (XRD) analysis shows the approximately 2 theta degree values of 38, 44, 64, and 77 obtained for both the HE-AgNPs (A) and HP-AgNPs (B), which correspond to the (111), (200), (220), and (311) planes of the face-centred cubic structure of silver NPs [48,49]. This affirmed the crystal nature of the particles. In addition, the crystallite size often calculated through this analysis usually support the size measured from microscopic analysis. The importance of these analyses is that the size and shape of nanoparticles dictate their application as with the HE-AgNPs and HP-AgNPs. The XRD analysis is often supported by selected area electron diffraction. Hence, a close examination of Figure 4C,D indicates bright circular rings which were attributed to the planes earlier reported by the XRD [50]. As a consequence, the two techniques gave evidence of the crystal properties of the nanoparticles.

### 2.5. Dynamic Light Scattering

**Figure 5:**
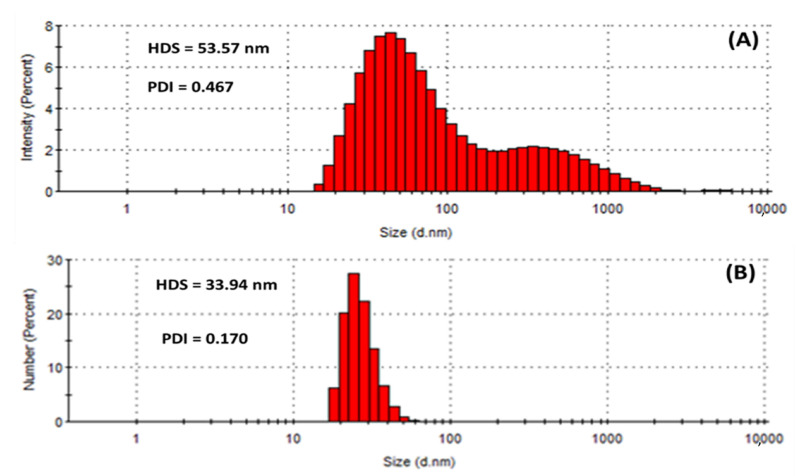
Hydrodynamic size (HDS) and polydispersity index (PDI) showing the population of particles in (**A**) HE-AgNPs and (**B**) HP-AgNPs.

Dynamic light scattering (DLS) measures the hydrodynamic size of colloids, which gives a rough estimate of the average size of the particles embedded in the solution. The hydrodynamic size (HDS) was found to be 53.57 nm and 33.94 nm for the HE-AgNPs and HP-AgNPs (Figure 5A,B), respectively. The results implied that the size of the HE-AgNPs is relatively larger than that of the HP-AgNPs. This might be explained by the number and type of constituents present in the reducing agents of the HE-AgNPs and HP-AgNPs. It is evident that while HE is a mixture of different phytochemicals, HP is a solution of hypoxoside bearing different functional groups. The difference in the HDS of the nanoparticles has been supported by both UV-Vis and TEM analysis, showing the agreement of the results. Additionally, larger particles scatter much more light than smaller ones. This may account for the larger HDS possessed by the HE-AgNPs over the HP-AgNPs. In colloidal solutions, larger particles are so dominating that even a small number can obscure the contribution of the smaller particles whatever their number. Nevertheless, it is noteworthy that the size measured by the DLS technique is often different from that of TEM [51]. This is because the size relates to the metallic core of the nanoparticles. The size is also influenced by all the substances surrounding the surface of the nanoparticles, such as the capping agents and the thickness of the solvation shell, moving along with the particles. The thickness of the solvation shell as well as its influence on the size of measured nanoparticles is, in turn, dependent on the nature of the substances in the colloidal suspension and on the surface of the nanoparticles. Hence, the size measured by the DLS technique was large than that measured by TEM [52]. Additionally, the difference in size is also due to the instrument used, since different instruments use specific operation techniques.

Similarly, the polydispersity index (PDI) of the HE-AgNPs (0.467) differs greatly from that of the HP-AgNPs (0.170) as provided in Figure 5. PDI is also known as the heterogeneity index. PDI is unitless. It is a measure of the non-uniformity of particles in each colloidal solution. Different algorithms have been used but the standard one considers values between 0.05 and 0.7 [53]. Colloidal solutions with PDI values close to 0.05 are deemed extremely monodispersed, meaning that almost a hundred percent of the particles are of the same shapes, whereas solutions with values close to 0.7 are considered heterogenous, implying that the colloid contains particles of different shapes. In light of this, it can be observed that the almost 0.5 PDI recorded is tending towards the other end of the standard scale (0.7), which means that the HE-AgNPs contain particles of various shapes. Additionally, the bimodal nature of the population (Figure 5A) also points to this fact. Conversely, the unimodal population in the case of the HP-AgNPs (Figure 5B) is justifiable by the PDI value of 0.170. The above findings are in good agreement with the results of UV-Vis, TEM, and HDS. Another key detail of the colloidal solution that can be obtained through DLS measurement is the zeta potential.

**Figure 6:**
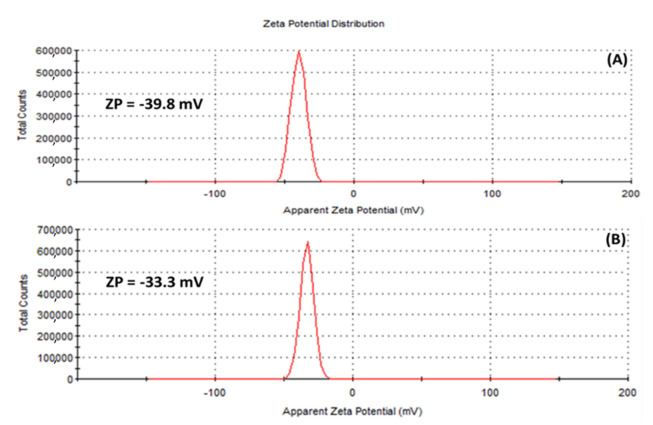
Zeta potential of (**A**) HE-AgNPs and (**B**) HP-AgNPs indicating that the surfaces of the nanoparticles are surrounded by negative ions.

The zeta potential (ZP), has to do with the charges surrounding the surface of nanoparticles and has been employed by many researchers to evaluate the stability of colloidal suspensions [54]. In this study, ZP values of −39.8 mV and −33.3 mV were obtained for the (A) HE-AgNPs and (B) HP-AgNPs, respectively (Figure 6), suggesting that stable silver nanoparticles were fabricated. Of interest is the fact that external stabilizers were not involved, meaning that the phytochemicals served both the purpose of reducing the silver ions to the silver of zero charge as well as stabilizing the silver nanoparticles upon formation. Previous studies have reported similar ZP values for silver nanoparticles [54].

### 2.6. Biological Activities

#### 2.6.1. Antimicrobial Studies of HE-AgNPs and HP-AgNPs

In a previous study, 50% methanol extract as well as petroleum ether extract of HE showed inhibitory effects against *Shigella flexneri* and *Trichophyton tonsurans* with MIC values of 780 and 390 µg/mL, respectively [19]. Similarly, *H. hemerocallidea* aqueous extract had an MIC value of 12.5 mg/mL against *S. aureus* and *E. coli* according to Ncube et al. [18]. The *H. hemerocallidea* aqueous extract also displayed an MIC value of about 480 µg/mL against *S. epidermidis*, *P. aeruginosa*, *S. aureus*, and *E. coli* [23]. The acetone corm extract of this plant also showed an MIC value of 310 µg/mL against *S. aureus* [48]. No report has shown the antimicrobial activity of hypoxoside alone to date, probably because of its low pharmacological properties. The antimicrobial efficacies of the HE-AgNPs and HP-AgNPs were evaluated against six different multi-drug resistant pathogenic bacteria using the microdilution assay. Bacterial species were divided into two groups: the Gram-positive strains consisting of *Bacillus cereus* and *Staphylococcus aureus* and the Gram-negative types which consist of *Escherichia coli*, *Pseudomonas aeruginosa*, *Salmonella enterica*, and *Serratia marcescens.* While the AgNPs were prepared in a concentration range of 125–1.95 µg/mL, the standard drug (ceftazidime) was set at the lowest concentration of the nanoparticles. At this concentration, the standard drug inhibited all the bacteria species and this was used to measure the efficacy of the AgNPs at various concentrations. Both the HE-AgNPs and HP-AgNPs showed growth inhibition of *S. aureus* at the highest tested concentration of 125 µg/mL. The second Gram-positive bacterium (*B. cereus*) was also moderately inhibited at the concentration of 62.50 µg/mL and 31.35 µg/mL by the HE-AgNPs and HP-AgNPs, respectively (Table 2). This seemingly weak inhibition when compared to the standard may be due to the nature of the bacterial species. Singh et al. have observed that Gram-positive bacteria species possess thicker cell wall peptidoglycan than the Gram-negative types. Therefore, penetration of the thick cell wall is difficult, resulting in reduced cell death [55]. Even so, the inhibition of *B. cereus* by the HP-AgNPs was still appreciable. This may be because of the small size of this nanoparticle. Previous studies have shown that AgNPs with small sizes have better bactericidal activities than those with larger sizes [41].

The Gram-negative bacteria species were appreciably inhibited by the AgNPs. *S. marcescens* and *P. aeruginosa* were inhibited to a similar extent by the two silver nanoparticles. The similarity of inhibition by the two different nanoparticles may be connected to the nature of the bacterial species and the mechanism of action of the bacteria. This, however, needs to be further understood as the exact mechanism of action is still in its infancy. A remarkable difference in the inhibition of *S. enterica* and *E. coli* was observed between the HE-AgNPs and HP-AgNPs (Table 2). This could be explained by two factors. One is the fact that these are Gram-negative species whose cell walls are made up of a thinner peptidoglycan layer, thereby allowing for easy cell disruption upon successful penetration. This might have caused the death of more cells [55]. Secondly, the size of the HP-AgNPs offers a better chance of penetration, thereby explaining why the inhibition was comparably higher than that of the HE-AgNPs [41].

Overall, the best activity from both the HE-AgNPs and HP-AgNPs was displayed against *E. coli*. While a good inhibition was recorded for the HE-AgNPs (31.25 µg/mL), the HP-AgNPs showed inhibition up to the least tested concentration (1.95 µg/mL), competing well with the standard drug used. This behaviour is supported by the literature [11]. The higher activity of the HP-AgNPs over the HE-AgNPs, in this case, might be due to the difference in size and morphology between the two. Previous evaluations have shown that AgNPs with smaller sizes possess better antibacterial activities when compared to those of larger sizes [40]. Alginate-mediated silver nanoparticles displayed distinct antimicrobial activity against *E. coli* with a very low MIC value. The authors attributed these activities to the small size and spherical nature of the AgNPs [41]. Also, when three polyphenols, namely naringin, hesperidin, and diosmin, were employed in silver nanoparticle formation, the naringin-AgNPs displayed a higher cytotoxicity than others which were linked to the small size and monodispersed nature of naringin-AgNPs compared to the large and polydispersed form of the AgNPs from hesperidin and diosmin [13]. Other researchers also showed that AgNPs between the sizes of 1–12 nm disrupted the external cell membrane of *E. coli* and caused cell death [56]. Hence, the smaller HP-AgNPs show predominantly higher activity than the HE-AgNPs. The smaller the size, the more the particles are able to penetrate the cell wall, which leads to more cell death. Taken together, the nanoparticles displayed better activities in the range of 125–1.95 µg/mL than the corresponding extracts (310 µg/mL–12.5 mg/mL) as reported in previous studies. This also confirmed that the antimicrobial activity of the nanoparticles was not due to the toxicity of the precursor reducing agents.

#### 2.6.2. Cytotoxic Activity of HE-AgNPs and HP-AgNPs

**Figure 7:**
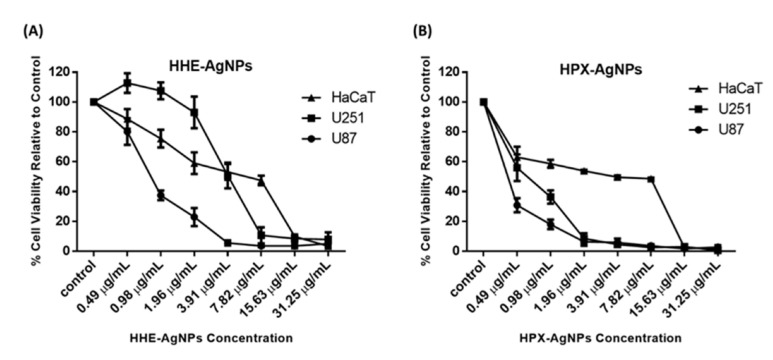
Cytotoxicity of HE-AgNPs (**A**) and HP-AgNPs (**B**) on the U251, U87, and HaCaT cell lines as obtained from MTT assays.

In a previous study, 50% methanol extract, as well as petroleum ether extract of HE, showed no cytotoxic effects against tested cell lines [19]. Similarly, the lack of genotoxic effects of water extracts of *Hypoxis hemerocallidea* was reported when human hepatoma HepG2 cells were used [19]. The cytotoxicity of hypoxoside alone has not yet been reported, probably due to its low pharmacological properties. In this study, the cytotoxic activities of HE-AgNPs and HP-AgNPs were investigated in the U251 and U87 brain tumour cell lines. The human skin keratinocytes (HaCaT) were used as a normal non-cancerous cell line as the human body is covered entirely by skin and as such will indicate the side effects of these nanoparticles. The MTT assay was used to assess cell viability in the cells after exposure to concentrations of the nanoparticles. The results show that both the HE-AgNPs and HP-AgNPs reduced cell viability in the U251 and U87 glioblastoma cell lines (Figure 7). Indeed, when the IC_50_ was calculated for the cells, the HE-AgNPs had an IC_50_ of 0.81 and 4.0 µg/mL, respectively, for the U87 and U251 cells while the HP-AgNPs displayed 0.20 and 0.55 µg/mL for both cell lines, respectively (Table 3). From the results, it is evident that the HP-AgNPs were more cytotoxic than the HE-AgNPs, which may indicate the activity of hypoxoside. The fact that the HP-AgNPs showed more bactericidal effects corroborates this finding. This fascinating activity of HP-AgNPs may be related to their size. Ultra-small-sized silver nanoparticles normally have the advantage of size in such a manner that penetration of cells is easier than for nanoparticles of larger sizes. It has been suggested that upon easy access to the cell, small-sized silver nanoparticles disrupt the external cell membrane, which often leads to cell death. Several previous studies have supported this submission [13,25,57].

Similarly, the activities of these nanoparticles were evaluated on HaCaT cells, and as observed in the glioblastoma cell lines, both nanoparticles reduced cell viability in the cells with an IC_50_ of 3.67 and 1.97 µg/mL for the HE-AgNPs and HP-AgNPs, respectively (Figure 7). Furthermore, the selectivity index of the HE-AgNPs and HP-AgNPs was calculated for both U87 and U251 cells for the HaCaT cells. The selectivity index is the ratio of IC_50_ in a normal cell to a cancerous one and an anticancer agent with a selectivity index greater than 2 is adjudged to be selective [58]. The results show that the HE-AgNPs had a selectivity of 4.53 and 0.92 µg/mL for U87 and U251, respectively, while the HP-AgNPs had a selectivity of 9.58 and 3.58 µg/mL, respectively (Table 3). From these results, it is evident that the HE-AgNPs were only selective to the U87 glioblastoma cells while the HP-AgNPs were selective to both glioblastoma cells tested. Taken together, these results indicate that both HE-AgNPs and HP-AgNPs induce cytotoxicity in the glioblastoma cells and that HP-AgNPs are overall more active as shown by the low IC_50_ and high selectivity index; this may warrant further investigation of HP-AgNPs as a potential anticancer agent in glioblastoma. Comparatively, 50% methanol extract as well as petroleum ether extracts of *Hypoxis hemerocallidea* a showed selectivity index of 21 and 19, respectively, in previous studies. This means that the reducing agents in the present study also have the potential to be developed into a safe anticancer agent [19].

## 3. Materials and Methods

### 3.1. Materials and Chemicals

Polystyrene 96-well microtitre plates were obtained from Greiner bio-one GmbH (Frickenhausen, BY, Germany). HPLC grade dichloromethane (DCM), methanol (MeOH), ethyl acetate (EtOAc), and silica gel 60 H of 0.040–0.063 nm particle size were purchased from Merck (Gauteng, Modderfontein, South Africa). Nuclear magnetic resonance (NMR) spectra were obtained using a Bruker spectrometer operating at 400 (for H)/100 (for C) MHz. Silver nitrate, Sephadex LH-20, iodonitrotetrazolium chloride (INT), ceftazidime (CTD), and sodium chloride (NaCl) were procured from Sigma-Aldrich (Cape Town, WC, South Africa). Phosphate buffered saline (PBS) was purchased from Lonza (Cape Town, WC, South Africa). Bacterial species (*Pseudomonas aeruginosa*, *Bacillus cereus*, *Staphylococcus aureus*, *Escherichia coli*, *Serratia marcescens*, and *Salmonella enterica)* were obtained from the Microbiology Laboratory, Department of Medical Biosciences, University of the Western Cape, South Africa. Brain–heart infusion broth (BHI) and Muller Hinton Agar were purchased from Biolab (Merck, Modderfontein, South Africa).

### 3.2. Isolation of Hypoxoside

The fresh bulb (374.0 g) was blended and extracted with methanol for 48 h. After filtration, the extract was concentrated under reduced pressure to give 97.20 g of a dark brown residue. About 90.0 g of this was subjected to silica gel column chromatography using a gradient of DCM and MeOH of increasing polarity. Subsequent purification of the hypoxoside-containing fraction was achieved after chromatography of the same fractions on Sephadex LH-20 using methanol and water in a 1:1 (*v*/*v*) ratio. The structure of the compound was confirmed using NMR spectroscopy, compared with the literature, and confirmed by co-spotting with an authentic sample in the laboratory.

### 3.3. Biofabrication of HE-AgNPs and HP-AgNPs

Before the synthesis, 30 mg each of HE and HP were suspended in 3 mL of ultra-pure deionized water and agitated until homogenous brownish and yellowish solutions were obtained, respectively. Thereafter, 2 mL of each aqueous solution was added to 100 mL of 1 mM silver nitrate (AgNO_3_) solution and heated at 60 °C with continuous stirring in the dark for 1 h. A change of colour to dark brown and pale yellow, respectively, indicated the formation of HE- and HP-AgNPs. The ultra-violet visible spectroscopic measurement of the biofabricated AgNPs was carried out with the aid of a plate reader (BMG Labtech, Ortenberg, Germany).

### 3.4. HRTEM Analysis

High-resolution transmission electron microscopy (FEI Tecnai G2 F20 S-Twin HRTEM, operating at 200 kV) was employed to study the morphology and crystallinity of the AgNPs. The size histogram of the nanoparticles was calculated using Image J software.

### 3.5. XRD Analysis

X-ray diffraction (XRD; Bruker AXS D8 advance diffractometer with CuKα1 radiation (λ = 1.5406 Å)) was employed to study the crystal structure of the biofabricated AgNPs.

### 3.6. Dynamic Light Scattering Analysis

A Malvern Zetasizer Instrument (Malvern Ltd., Malvern, UK) operating at angles of 25 and 90 degrees was used to obtain information on the hydrodynamic size, polydispersity index, and zeta potential of the AgNPs. Solutions of HE-AgNPs and HP-AgNPs were measured in disposable quartz cuvettes upon cooling to room temperature.

### 3.7. Antimicrobial Activity

The method described by Shao et al. [41] was used with slight modification. Briefly, brain–heart infusion broth (BHI) was employed for serial dilution of both HE- and HP-AgNPs into 125.00, 62.50, 31.25, 15.63, 7.81, 3.90, and 1.95 µg/mL. Bacterial species in PBS were standardized to 0.5 McFarland equivalent and further diluted in BHI. Three controls were used: 200 µL of BHI, 200 µL of sterilized deionized water, and a mixture of 100 µL of the test organism and 100 µL of sterilized deionized water. After 24 h of incubation, 40 µL of INT was added and sealed to avoid evaporation and then incubated for 2 h, after which examination for a colour change took place. A clear BHI-like colouration indicates the death of bacteria, whereas a turbid or pinkish colour shows growth. The MIC refers to the lowest concentration of AgNPs that completely inhibits bacterial growth. All tests were done in triplicate. Ceftazidime, which was prepared in the same manner as the AgNPs, was employed as a positive control. The selected bacteria were wild types.

### 3.8. Cell Culture and Maintenance

The World Health Organization grade IV human malignant glioma cell lines U251 (ECACC 09063001, European Collection of Authenticated Cell Cultures) and U87 (ATCC HTB-14, American Type Culture Collection) were generously donated by the Prince Laboratory, Faculty of Health Sciences, University of Cape Town, Cape Town (Omoruyi et al., 2020), while the human keratinocyte cell line HaCaT (used as a normal non-cancerous cell line) (Boukamp et al., 1988) was a generous donation by the Mintek Laboratory, Department of Biotechnology, University of the Western Cape. Cells were cultured in monolayer using Dulbecco’s modified Eagle’s medium (DMEM, Lonza Group Ltd., Verviers, Belgium) supplemented with 10% foetal bovine serum (FBS, Gibco, Life Technologies Corporation, Paisley, UK) and 1% 100 U/mL penicillin and 100 μg/mL Streptomycin (Lonza Group Ltd., Verviers, Belgium). All cells were grown at 37 °C, in a humidified atmosphere consisting of 5% CO_2_ and 95% air. All cell culture media were routinely replaced every two to three days and were subcultured when they attained 80% confluence using a solution of 25% trypsin EDTA (Lonza Group Ltd., Verviers, Belgium). A mycoplasma test was also conducted at intervals and only cells that were mycoplasma-free were used for experiments [59,60].

Cell Viability Assay

Cell viability was assessed using the 3-[4,5-dimethylthiazol-2-yl]-2,5diphenyltetrazolium bromide (MTT, Sigma-Aldrich, St. Louis, MO, USA) colourimetric assay. The MTT is a yellow tetrazolium salt which is reduced to purple formazan in living cells. Briefly, the U251 and U87 glioblastoma cells as well as the HaCaT keratinocytes were plated in 96-well cell culture plates at a cell density of 5000 cells per well and incubated overnight to allow for attachment. The medium was thereafter replaced with fresh medium containing increasing concentrations (0.49 µg/mL, 0.98 µg/mL, 1.96 µg/mL, 3.91 µg/mL, 7.82 µg/mL, 15.63 µg/mL, and 31.25 µg/mL) of HE-AgNPs and HP-AgNPs for 48 h. Cells incubated with medium only served as control. After the 48 h incubation, 10 μL of the MTT solution (5 mg/mL) was added to each well and the cells were incubated for an additional 4 h at 37 °C. Thereafter, the resultant MTT crystals formed were solubilized in dimethyl sulfoxide (DMSO, Sigma-Aldrich, St. Louis, MO, USA) and absorbance of each well was read at 570 nm using a BMG Labtech Omega^®^ POLARStar multimodal plate reader. The percentage cell viability was calculated using the formula below:

$$\%\;\mathrm{Cell}\;\mathrm{Viability}=\frac{\mathrm{Absorbance}\;\mathrm{of}\;\mathrm{treated}\;\mathrm{well}}{\mathrm{Absorbance}\;\mathrm{of}\;\mathrm{Untreated}\;\mathrm{well}}\times 100$$

The concentration required to kill 50% of the cells (IC_50_) was determined via a survival curve using GraphPad Prism6 software (GraphPad software, San Diego, CA, USA) and selectivity index (ratio of IC_50_ in a normal cell to a cancer cell) was calculated. All experiments were conducted in triplicate.

## 4. Conclusions

A facile, cost-effective, and eco-friendly protocol led to the biofabrication of HE- and HP-AgNPs. The AgNPs were fully characterized using UV-Vis, TEM, SAED, HDS, PDI, ZP, and XRD. The small-sized AgNPs obtained were found to possess antimicrobial and cytotoxic activities. The study demonstrated, for the first time, that the constituents of HE possess the required functionality to aid the successful green synthesis of silver nanoparticles. The study also shows the involvement of hypoxoside as a single phytochemical serving the purpose of reduction and stabilization in both HE- and HP-AgNPs. Because of its unique characteristics, the smaller, spherical, monodispersed, and stable HP-AgNPs displayed better antimicrobial and cytotoxic activities against *E. coli* and *S. enterica*. They also displayed a better reduction of the glioblastoma cells tested over the HE-AgNPs. Overall, better antimicrobial activities were displayed by the nanoparticles when compared to the inhibitory activities of their precursor plant extracts in previous studies. Further studies on the activity of hypoxoside (a pharmacologically inactive compound) are required, as converting it to another form seems to give promising results.

## Figures and Tables

**Table 1:** HE-AgNPs consist of various shapes with spheres dominating.

Nanoparticles	Shape Distribution (%)		
	Oval	Cone	Cylinder
HE-AgNPs	84.01	0.93	15.06

**Table 2:** MIC values of the antimicrobial activities of AgNPs and the standard in µg/mL.

Antimicrobial Activities of Silver Nanoparticles			
Bacteria Species	HE-AgNPs	HP-AgNPs	CTD
*S. aureus*	125.00	125.00	1.95
*B. cereus*	62.50	31.35	1.95
*S. enterica*	125.00	1.95 **	1.95
*S. marcescens*	125.00	125.00	1.95
*E. coli*	31.25	1.95 **	1.95
*P. aeruginosa*	62.50	62.50	1.95

CTD: Ceftazidime, HE-AgNPs: Hypoxis hemerocallidea extract-mediated silver nanoparticles, HP-AgNPs: hypoxoside-mediated silver nanoparticles. **: MIC values equal to those of the standard drug used.

**Table 3:** IC_50_ values and selectivity index (SI) of AgNPs.

NPs Samples	Test Items	U87	U251	HaCaT
		(µg/mL)	(µg/mL)	(µg/mL)
HE-AgNPs	IC50	0.81	4.0	3.67
	SI	4.53	0.92	
HP-AgNPs	IC50	0.20	0.55	1.97
	SI	9.85	3.58	

## Data Availability

Publicly available datasets were analyzed in this study. This data can be found here: [http://etd.cput.ac.za/bitstream/20.500.11838/3188/1/Umar_Muhammed_217064221.pdf, 1 February 2022].
